# Persistent Genital Arousal Disorder: Two Case Studies and Exploration of a Novel Treatment Modality

**DOI:** 10.1089/whr.2022.0097

**Published:** 2023-02-14

**Authors:** Meghan Scantlebury, Romeo Lucas

**Affiliations:** ^1^ University of New England College of Osteopathic Medicine, Biddeford, Maine, USA.; ^2^ New England Center for Pelvic Health, Freeport, Maine, USA.

**Keywords:** persistent genital arousal disorder, pelvic pain, sexual dysfunction

## Abstract

Persistent genital arousal disorder (PGAD) is characterized by persistent unwanted feelings of sexual arousal that can be debilitating. Despite first being defined >20 years ago, the precise etiology and treatment of this disorder remain elusive. Mechanical disruption of nerves, neurotransmitter changes, and cyst formation have all been considered as etiologies involved with the development of PGAD. With limited and ineffective treatment modalities, many women live with their symptoms untreated or undertreated. To broaden the literature, we present two cases of PGAD and present a novel treatment modality of the disorder with the use of a pessary. Although there was subjective success in dampening the symptoms, they were not completely resolved. These findings open the door for the potential of similar treatments in the future.

## Introduction

Persistent genital arousal disorder (PGAD) was first described by Leiblum and Nathan in 2001 in a five-patient case series. Initially classified as “persistent sexual arousal syndrome,” the diagnosis referred to “sensations of sexual arousal [that] are persistent, insistent, and unrelated to conscious feelings of desire,” and that may or may not be associated with an identifiable trigger.^1^ This diagnosis has been renamed PGAD and was further expanded to include genitopelvic dysesthesia (GPD) to better encompass the variety of symptoms that may be experienced with this disorder, aside from persistent arousal.^2^

Case reports continue to be published throughout the literature; however, due to the nature of the condition, there has been difficulty in determining its prevalence. Limited estimations complied from a variety of sources with populations from different countries estimate that the prevalence ranges from 0.6% to 3%.^2^

Given the majority of information surrounding PGAD/GPD is from case reports, diagnosis and treatment continue to be a challenge. During their most recent meeting in 2019, the International Society for the Study of Women's Sexual Health (ISSWSH) introduced a novel management algorithm to better categorize and determine the source of symptoms patients with PGAD experience. The five regions that this panel decided warranted consideration are end organ, pelvis/perineum, cauda equina, spinal cord, and the brain.^2^ The suggestion was that after determining the patient meets criteria for PGAD, the clinician should focus attention on the location provoking their symptomology to direct their treatment.^2^

Current hypotheses indicate that disruptions in nerve pathways, either through mechanical interference, cyst formation, or neurotransmitter changes, lead to sensory changes that present as PGAD symptoms.^2,3^ The female reproductive system carries somatic, parasympathetic, and sympathetic innervation. Somatic innervation is derived mainly from the pudendal nerve, originating from the sacral plexus at the S2–S4 levels.^4^ Sympathetic innervation to the female genitalia originates from the lower thoracic and upper lumbar spinal segments, typically T11 to L2.^5^ Parasympathetic fibers arise from the pelvic splanchnic nerves at the S2–S4 segments.^4^ Ultimately, the parasympathetic and sympathetic nerves combine to form the inferior hypogastric plexuses in the pelvis.

PGAD resulting from cauda equina-derived pathology could be secondary to a radiculopathy of the sacral spinal nerve roots.^2^ Another well-documented source is from cerebrospinal fluid-filled aneurysm-like Tarlov cysts, which typically form at, or just distal to, the dorsal root ganglia on sacral nerves.^6^ The pressure from these cysts has the potential to irritate nerve fibers, including those that are destined to innervate the genitalia.^2,6^ Other potential sources include lumbar and lumbosacral intervertebral disk pathologies, including disk herniation and spinal stenosis, which also have the potential to irritate the nerve fibers of the pudendal nerve proximally, resulting in the sensations that are felt with PGAD.^7^

Distinguished as a separate etiology, spinal cord pathology may also bring about the symptoms of PGAD. Trauma and injuries falling within this category would impact mainly the thoracic or cervical spinal cord, above the termination of the spinal cord at the L1/L2 level. Pathologies that may impact the spinal cord are similar to those that may impact the cauda equina, including annular tears, disk herniation, and spinal stenosis.^2,6^

In addition to disturbance of the nerve pathways between the brain and genitalia, there are also hypotheses involving other neurotransmitter abnormalities as a cause of PGAD. It is known that cognitive, neurological, hormonal, and biochemical components each play a vital role in interpreting and producing the changes that are ultimately perceived by the individual to represent sexual arousal. Sexual arousal resulting from direct touch of the genitalia is not transmitted along one specific type of nerve fiber, but is instead likely due to integration of both small and large diameter sensory fibers.^8^

Both the small and large fiber nerves transmit their sensory information to the spinal cord where they are attenuated in the substantia gelatinosa and ultimately interpreted as sexual arousal and pleasure by higher level processing centers in the brain.^9^ Descending inhibitory pathways from the central nervous system that attenuate the afferent peripheral sensory signals utilize both serotonin and norepinephrine as neurotransmitters. Experiments have shown that increased norepinephrine or serotonin in the spinal cord can produce analgesic effects and increase the nociceptive threshold.^10^ Studies have also shown that vaginal stimulation results in an increased pain tolerance.^11^

Treatment for PGAD continues to be a poorly understood area that has, up to this point, been based almost entirely upon case reports and expert opinion. There are currently no medications that are approved for the treatment of PGAD, thus treatment is carried out with off-label uses of medications or devices targeting the most likely regional etiology of the patient's PGAD. Current medications and modalities that have been used with varying success include gabapentin, pregabalin, antidepressants, anticonvulsants, benzodiazepines, electroconvulsive therapy, lumbar disk disease surgery, dose adjustments of selective serotonin reuptake inhibitors/selective serotonin-norepinephrine reuptake inhibitors, pudendal nerve blocks, topical anesthetics, and leuprolide.^2,12,13^

## Patient 1

N.L. is a 67-year-old G3P2012 woman, now postmenopausal, with a medical history that includes bipolar 1 disorder managed initially with electroconvulsive therapy and now maintained on trazodone and lamotrigine; type 2 diabetes mellitus on metformin and semaglutide; hypertension on losartan; hyperlipidemia on rosuvastatin; depression; anxiety; and chronic back pain after a motor vehicle accident at age 20 years who presented as a new patient referral to the gynecology office for PGAD. The onset of her symptoms initially began just weeks after an L4–L5 lumbar laminotomy at age 55 years.

Per patient, the symptoms began as a “pressure-like” feeling to her genitalia, as if she were wearing underwear that were too tight. She now describes having a sensation of spam, “throbbing, aching, and pounding pain,” and feeling as if she is on the verge of orgasm, although she does deny any spontaneous orgasms associated with her symptoms. She reports that the pain she feels can be significant, at times rating it a 10/10 in severity. Her symptoms are brought on by lying and sitting, but are not present when walking or standing. Her symptoms are also reported to be exacerbated by stress. She attempts to control her pain by sitting on ice for prolonged periods of time throughout the day.

One year after her initial L4–L5 laminotomy, the patient suffered from a fall with subsequent difficulty walking secondary to pain and required a revision of the laminotomy at the L4–L5 level. At the age of 64 years, she underwent an additional laminotomy at the L5–S1 level. The patient reports that she continues to have lumbar back pain and sciatica.

The patient had previously seen a provider who had administered a pudendal nerve block, which the patient reports did not resolve her symptoms, and had started the patient on gabapentin 100 mg TID. During her initial new patient examination, the patient reported having decreased sensation of arousal and of her symptoms when two digits put pressure on the posterior fornix. Maintaining this pressure results in a decrease of both her arousal and the throbbing painful pressure that the patient experienced.

Based on this finding, the decision was made to place a pessary to maintain pressure on the vaginal walls in an attempt to decrease her symptoms. Additional initial treatment included increasing her gabapentin dose to 600 mg TID and providing a vaginal valium cream every other day to provide muscle relaxation and symptom relief. The patient notes that when she misses a dose of the gabapentin, she has a worsening of her PGAD symptoms.

Upon follow-up the ∼2 weeks later, the patient reported only minimal improvement with the new additions of vaginal diazepam cream and the pessary; however, she felt that she wanted to continue with these treatments for a while longer and continue to monitor.

A few months later, the patient continued to feel there was no significant improvement with the pessary and the decision was made to discontinue this treatment. The vaginal diazepam and gabapentin were continued, as the patient felt that she did have some mild improvement with these interventions. Shortly after, however, the patient made the decision to discontinue the vaginal diazepam and was then started on a vaginal suppository of 10 mg of ketamine and 20 mg of amitriptyline daily. The patient continues to report that these suppositories are providing her with some relief from her symptoms; however, they have not fully resolved. She also continues to take gabapentin 600 mg TID. She is currently pursuing neurostimulation therapy as a definitive intervention.

## Patient 2

Patient 2 is a 55-year-old female with a medical history of asthma, degenerative disk disease (cervical and lumbosacral), depression on bupropion, hypertension, obesity, and prior motor vehicle accident at age 27 years with resultant neck and back injury who presented after referral from an outside provider. The patient reports that she feels she is constantly aroused and has hypersensitivity of the clitoris and vulvar pain, making it hard for her to sit still for prolonged periods of time. She remembers having these symptoms as far back as her teenage years, but is unsure of the precise age of onset. She recalls that she did not wear pants until high school, opting for loose fitting clothing, such as dresses and skirts.

Her symptoms have been increasingly more intrusive after menopause at age 50 years. Her persistent arousal is made worse when wearing tight fitting clothing. When she focuses on other tasks, such as knitting, writing, and reading, she is able to achieve some relief. Standing and applying firm pressure over the vulva also somewhat improves her symptoms. To fall asleep at night, the patient places very cold wash clothes over her vulva.

Before her initial presentation, the patient had tried physical therapy, vaginal dilation, botulinum toxin muscle injection, pudendal nerve block, benzocaine-lidocaine-tetracaine ointment, and cognitive behavior therapy, all of which were not helpful in resolving her persistent arousal. The patient did have some improvement of her localized vulvar pain with botulinum toxin muscle injections and estradiol ointment; however, the feelings of sexual arousal persisted. At her time of initial presentation, she was taking gabapentin 100 mg in the morning and 300 mg at night, which she has been using for 2 years.

She also reports using calmoseptine ointment multiple times throughout the day. The patient also trialed stopping her bupropion, but reports that it did not make a difference in her symptoms. The patient has had a prior MRI that did not show any masses or cysts along the course of the pudendal nerve and reported nothing notable about the sacral plexus.

The patient was trialed on carbamazepine briefly, which was found to cause some improvement in her symptoms; however, it made her dizzy, which interfered with her daily life, and was discontinued. She had a pessary inserted, which was reported to provide improvement from her symptoms for ∼1 week, after which time she felt the symptoms begin to return. The patient was trialed off the pessary, as she did not feel that it was improving her symptoms any longer; however, after it was removed, she had a worsening of her symptoms and requested that it be replaced. She had additional improvement with daily vaginal diazepam.

## Discussion

Both patients presented emulate the classic symptoms of PGAD and presented to the office having undergone multiple treatments, including pudendal nerve blocks. In these cases, the patients did not have resolution of their symptoms after the nerve blocks, suggesting a more proximal etiology of their pain, such as the cauda equina, spinal cord, or brain.^2,6^ Thought to be a common cause of PGAD originating at the level of the cauda equina are Tarlov cysts, which increase pressure on the nerve root at which they originate and have the potential to disrupt surrounding nerve roots as well.^3^

For these patients, Tarlov cysts are less likely given their imaging, which did not demonstrate any lesions or cysts. The first case presents an extensive history of back pain after involvement in a car accident many years ago, now with ongoing back pain focal to her lumbosacral area. She additionally includes history that her symptoms seemed to occur a few weeks after her lumbar laminotomy. This is most consistent with a cauda equina-derived etiology of her PGAD, which may have been secondary to disruption of the cauda equina during or after her surgery.

The second patient also noted a history of spinal pathology, endorsing a history of degenerative disk disease in the cervical and lumbosacral spine. Given that her symptoms began early in her life, before the onset of her back pain, and worsened at the time of menopause, it is unlikely that her known history of degenerative disk disease plays a major role in the particular etiology of her PGAD.

Multiple treatment modalities were highlighted as part of this case series. Both patients were trialed on vaginal diazepam and did show initial improvement. Patient 2 continues to have improved symptoms with vaginal diazepam, whereas patient 1 ultimately discontinued vaginal diazepam to use a combination of 10 mg of ketamine and 20 mg of amitriptyline suppository.

Diazepam is a benzodiazepine that works by binding gamma-aminobutyric acid type A receptors to allow for increased flow of chloride through the receptor, resulting in an inhibitory effect.^14^ It has been documented as treatment for PGAD and for other disorders, such as vulvodynia and vestibulodynia. In addition to exerting inhibitory effects at the level of the conus medullaris, it may also serve to decrease muscle tightness or spasm, which may contribute to feelings of arousal.^2,15^

Compounded amitriptyline–ketamine was also used to treat patient 1. It is hypothesized that these medications are able to attenuate unpleasant stimuli. Amitriptyline is thought to function as a sodium channel blocker when used topically and ketamine primarily blocks N-methyl-D-aspartate receptors in peripheral nerves.^16^

In this case series, we also presented a novel treatment for PGAD with the use of a pessary. The insertion of a pessary is thought to exert vaginal stimulation, in the form of pressure, onto the vaginal walls, in an attempt to produce analgesic effect and lessen the symptoms of PGAD. It is known that vaginal stimulation in rats demonstrates an increased analgesic effect after exposure to a noxious stimuli.^10^ This has been extrapolated to human studies that also demonstrated vaginal stimulation increasing the threshold for pain.^11^

Patient 1 felt as if the pessary was not adequately contributing to her symptom relief and decided to discontinue its use. Patient 2 reported some improvement of her symptoms with the pessary in place. At one point, she was trialed off the pessary; however, she felt that her symptoms worsened without it and did have the pessary replaced.

The initial improvement that both patients endorsed with the pessary inserted was encouraging, even though patient 1 did ultimately decide against the use of the pessary. Having some benefit from the pessary supports the potential utility of an intravaginal device working to provide vaginal stimulation, in the form of pressure, to elicit an inhibitory effect on their symptoms.

## Abbreviations Used

GPD: genitopelvic dysesthesia
ISSWSH: International Society for the Study of Women's Sexual Health
PGAD: persistent genital arousal disorder

## References

[1] Leiblum SR, Nathan SG. Persistent sexual arousal syndrome: A newly discovered pattern of female sexuality. J Sex Marital Ther 2001;27(4):365–380; doi: 10.1080/009262301317081115 11441520

[2] Goldstein I, Komisaruk BR, Pukall CF, et al. International Society for the Study of Women's Sexual Health (ISSWSH) Review of Epidemiology and Pathophysiology, and a Consensus Nomenclature and Process of Care for the Management of Persistent Genital Arousal Disorder/Genito-Pelvic Dysesthesia (PGAD/GPD). J Sex Med 2021;18(4):665–697; doi: 10.1016/j.jsxm.2021.01.172 33612417

[3] Oaklander AL, Price BH, Sharma S, et al. Persistent genital arousal disorder: A special sense neuropathy. Pain Rep 2020;5(1):E801; doi: 10.1097/PR9.0000000000000801 32072096 PMC7004503

[4] Shoja MM, Sharma A, Mirzayan N, et al. Neuroanatomy of the female abdominopelvic region: A review with application to pelvic pain syndromes. Clin Anat 2013;26(1):66–76. 23175283 10.1002/ca.22200

[5] Purves D, Augustine GJ, Fitzpatrick D, et al. Autonomic regulation of sexual function. In: Neuroscience, 2nd edition. Sinauer Associates: Sunderland, MA; 2001.

[6] Komisaruk BR, Lee H. Prevalence of sacral spinal (Tarlov) cysts in persistent genital arousal disorder. J Sex Med 2012;9(8):2047–2056; doi: 10.1111/j.1743-6109.2012.02765.x 22594432

[7] Komisaruk BR, Goldstein I. Persistent genital arousal disorder: Current conceptualizations and etiologic mechanisms. Curr Sex Health Rep 2017:1–6; doi: 10.1007/s11930-017-0122-5

[8] Vilimas PI, Yuan S, Haberberger RV, et al. Sensory innervation of the external genital tract of female guinea pigs and mice. J Sex Med 2011;8(7):1985–1995; doi: 10.1111/j.1743-6109.2011.02258.x 21477025

[9] Martin-Alguacil N, Schober JM, Sengelaub DR, et al. Clitoral sexual arousal: Neuronal tracing study from the clitoris through the spinal tracts. J Urol 2008;180(4):1241–1248; doi: 10.1016/j.juro.2008.06.009 18707740 PMC2740385

[10] Steinman JL, Komisaruk BR, Yaksh TL, et al. Spinal cord monoamines modulate the antinociceptive effects of vaginal stimulation in rats. Pain (Amsterdam) 1983;16(2):155–166; doi: 10.1016/0304-3959(83)90205-1 6877846

[11] Whipple B, Komisaruk BR. Elevation of pain threshold by vaginal stimulation in women. Pain 1985;21(4):357–367; doi: 10.1016/0304-3959(85)90164-2 4000685

[12] Cohen SD. Diagnosis and treatment of persistent genital arousal disorder. Rev Urol 2017;19(4):265–267; doi: 10.3909/riu0784 29472831 PMC5811885

[13] Deka K, Dua N, Kakoty M, et al. Persistent genital arousal disorder: Successful treatment with leuprolide (antiandrogen). Indian J Psychiatry 2015;57(3):326–328. 26600597 10.4103/0019-5545.166633 PMC4623662

[14] Stone RH, Abousaud M, Abousaud A, et al. A systematic review of intravaginal diazepam for the treatment of pelvic floor hypertonic disorder. J Clin Pharmacol 2020;60:S110–S120; doi: 10.1002/jcph.1775 33274514

[15] Murina F, Felice R, Di Francesco S, et al. Vaginal diazepam plus transcutaneous electrical nerve stimulation to treat vestibulodynia: A randomized controlled trial. Eur J Obstetr Gynecol Reprod Biol 2018;228:148–153; doi: 10.1016/j.ejogrb.2018.06.026 29960200

[16] Poterucha TJ, Murphy SL, Rho RH, et al. Topical amitriptyline-ketamine for treatment of rectal, genital, and perineal pain and discomfort. Pain Physician 2012;15(6):485–488. 23159965

